# Integrated Rumen Metabolomics and Metagenomics Reveal Microbe–Metabolite Signatures Associated with Heat Tolerance in Dairy Cows

**DOI:** 10.3390/ani16142152

**Published:** 2026-07-11

**Authors:** Peng Chen, Can Liu, Shimeng Wang, Hao Zhang, Jiaxi Li, Niel A. Karrow, Yongjiang Mao, Zhangping Yang, Mingxun Li

**Affiliations:** 1 Key Laboratory of Animal Genetics & Breeding and Molecular Design of Jiangsu Province, College of Animal Science and Technology, Yangzhou University, Yangzhou 225009, China; mx120240895@stu.yzu.edu.cn (P.C.); lc1714543081@163.com (C.L.); mz120241629@stu.yzu.edu.cn (S.W.); mx120250916@stu.yzu.edu.cn (H.Z.); mx120520941@stu.yzu.edu.cn (J.L.); cattle@yzu.edu.cn (Y.M.); yzp@yzu.edu.cn (Z.Y.); 2 Center for Genetic Improvement of Livestock, Department of Animal Biosciences, University of Guelph, Guelph, ON N1G 2W1, Canada; nkarrow@uoguelph.ca

**Keywords:** dairy cows, heat stress, rumen metabolomics, microbe–metabolite interaction, biomarkers

## Abstract

Heat stress impairs dairy cow productivity and rumen function, but rumen metabolic features associated with natural heat tolerance remain unclear. This study generated rumen fluid metabolomic data and integrated them with previously generated metagenomic abundance data from the same heat-tolerant (HT) and heat-sensitive (HS) Holstein cows selected from a cohort of 120 cows using an entropy-weighted TOPSIS model. Untargeted LC-MS identified 116 differential metabolites, including 66 enriched in HS cows and 50 enriched in HT cows. The HS cows showed higher levels of nucleotide-related metabolites, whereas HT cows were enriched in thiamine, L-malate, and argininosuccinic acid. Pathway enrichment mainly involved nucleotide metabolism, pyrimidine metabolism, pyruvate metabolism, and thiamine metabolism. Reanalysis of metagenomic data identified 12 differential microbial taxa, including HT-enriched *Prevotella* and *Ruminococcus flavefaciens*. Spearman correlation analysis revealed phenotype-associated microbe–metabolite associations, and ROC analysis based on the discovery dataset suggested that uridine 5′-monophosphate, thiamine, L-malate, and argininosuccinic acid had exploratory potential to distinguish HT and HS cows. These findings provide exploratory evidence that rumen microbe–metabolite associations are related to natural heat tolerance in dairy cows.

## Simple Summary

Hot weather can reduce milk production and disturb digestion in dairy cows, but some cows cope better than others. In this study, we compared rumen fluid from cows that were either more tolerant or more sensitive to natural summer heat. We combined measurements of rumen fluid metabolites with previously generated information on rumen microorganisms from the same animals. The heat-sensitive cows showed higher levels of molecules related to nucleotide metabolism, whereas the heat-tolerant cows had higher levels of thiamine, L-malate, and argininosuccinic acid. Some rumen microorganisms were also linked with these metabolic differences. These findings suggest that the rumen microbial and metabolic environment may be associated with natural heat tolerance in dairy cows. The results provide preliminary candidate indicators that may help future studies improve heat tolerance evaluation and breeding strategies.

## 1. Introduction

Heat stress is a major environmental challenge to dairy production worldwide, particularly for high-yielding lactating cows with substantial metabolic heat output. Prolonged exposure to high temperature and humidity disrupts thermoregulation, depresses feed intake, impairs rumen fermentation, reduces milk yield, and compromises immune and metabolic homeostasis [1,2,3]. These effects not only threaten animal welfare and production efficiency but also pose increasing risks to sustainable dairy systems under changing climate conditions [1,2,3,4].

Dairy cows exhibit considerable individual variation in their responses to the same thermal environment. Under comparable feeding and management conditions, some cows maintain relatively stable body temperature, physiological status, and production performance, whereas others develop pronounced heat-sensitive phenotypes. This variation indicates that heat tolerance is a complex adaptive trait shaped by coordinated regulation of thermoregulatory capacity, metabolic resilience, immune resilience, and host–microbiome interactions [5,6]. Therefore, defining the biological features that distinguish heat-tolerant (HT) from heat-sensitive (HS) cows is essential for precision phenotyping and the development of biomarker-assisted selection strategies [7].

The rumen is a central hub of host–microbe co-metabolism in dairy cows. Through the degradation of structural carbohydrates, protein turnover, nitrogen recycling, and volatile fatty acid production, rumen microorganisms provide critical substrates for host energy supply and metabolic stability and also generate bioactive metabolites involved in host metabolic and immune regulation [8,9,10]. Heat stress can perturb this ecosystem by altering feed intake, rumination behavior, saliva buffering, fermentation patterns, and microbial community structure [5,6,11]. Consequently, rumen microbial composition and metabolic activity may be key determinants of divergent adaptive responses to thermal stress [6,11].

Our previous rumen metagenomic study, which was based on the same heat-stress cohort used in the present study, revealed distinct microbial compositions and functional potentials between HT and HS cows, suggesting that rumen microbial ecology is closely linked to natural thermotolerance [7]. However, taxonomic and functional profiles alone cannot fully explain the contribution of rumen microorganisms to host adaptation, because microbial effects are ultimately mediated via their metabolic products. Rumen metabolites represent the direct biochemical output of host–microbe co-metabolism and can reflect shifts in energy metabolism, nucleotide turnover, cofactor availability, nitrogen metabolism, oxidative stress responses, and immune function [10,12,13]. Therefore, integrating rumen metabolomics with microbial abundance data is necessary to link microbial variation with metabolic consequences relevant to heat tolerance [12,13].

In this study, we characterized rumen metabolic signatures and microbe–metabolite interaction patterns associated with heat tolerance in dairy cows. Using HT and HS cows previously identified by an entropy-weighted TOPSIS model, we performed untargeted metabolomic profiling of rumen fluid, and integrated these data with metagenomic abundance information from the same animals [7]. We aimed to identify differential metabolites, enriched metabolic pathways, key microbial taxa, and representative microbe–metabolite associations related to thermotolerance. This study provides new insight into the rumen microecological and metabolic basis of thermal adaptation and identifies candidate metabolic indicators for future validation in heat-tolerant dairy cattle.

## 2. Materials and Methods

### 2.1. Animals, Grouping, and Sample Source

The animal experiment, heat tolerance classification, and rumen fluid sampling procedures were based on our previous rumen metagenomic study [7]. Briefly, the trial was conducted from July to September 2023 at Weihang Farm in Suqian, Jiangsu Province, China. A total of 120 high-producing Chinese Holstein cows were enrolled and evaluated under natural summer heat stress conditions. The enrolled cows had an average parity of 2.21 ± 0.79, days in milk of 206.71 ± 54.29 days, and daily milk yield of 34.21 ± 18.09 kg, as reported in our previous study [7]. To minimize environmental and management-related variation, all cows were housed in the same barn equipped with fans and an automatic sprinkler system. The same 30 min cooling procedure was applied to all cows before each milking. All cows were fed the same total mixed ration (TMR) 3 times daily at 6:30, 14:30, and 20:00, with free access to feed and water.

Heat tolerance was evaluated using an entropy-weighted TOPSIS model as described in our previous study [7]. Briefly, environmental temperature and relative humidity were recorded at 5 min intervals during the trial, and the temperature–humidity index (THI) was calculated to characterize heat-stress exposure. During the assessment period, the daily average THI was 81.1 ± 3.17, indicating sustained summer heat-stress conditions. Physiological indicators, including rectal temperature (RT), respiratory rate (RR), and salivation index (SI), were measured daily between 13:00 and 15:00, corresponding to the peak daytime heat-stress period. Milk yield was recorded daily, whereas serum potassium ion (K^+^), heat shock protein 70 (HSP70), and cortisol levels were measured from blood samples collected on day 25 of the trait measurement period. These physiological, production, and serum biochemical indicators were integrated using the entropy-weighted TOPSIS model to classify cows with contrasting heat tolerance phenotypes [7]. Based on the comprehensive heat tolerance scores, cows ranking in the top 5% were classified as heat-tolerant (HT), whereas those in the bottom 5% were classified as heat-sensitive (HS). The detailed TOPSIS scores and phenotypic differences between the selected groups have been reported previously [7]. Six HT and six HS cows were selected for the present rumen metabolomic and integrative analyses. The animal experiment was approved by the Animal Ethics Committee of Yangzhou University, with protocol number 202304032 approved on 8 April 2023.

### 2.2. Rumen Fluid Collection and Processing

Rumen fluid samples were collected after heat tolerance classification. Briefly, samples were obtained from the selected HT and HS cows using a rumen tube on d 50 of the trial. To reduce feed particle contamination, rumen fluid was filtered through 4 layers of sterile gauze immediately after collection. The filtrate was aliquoted into sterile cryo-vials, snap-frozen in liquid nitrogen, and stored at −80 °C until untargeted metabolomic analysis. The same rumen fluid samples were previously used for metagenomic sequencing [7]; therefore, the present study focused on rumen metabolomic profiling and integration of these data with the existing metagenomic abundance data.

### 2.3. Untargeted Metabolomic Profiling and Data Processing

Untargeted metabolomic profiling of rumen fluid was performed by Shanghai Lin-gen Biotechnology Co., Ltd. (Shanghai, China). Briefly, rumen fluid samples were thawed on ice, and an aliquot of each sample was extracted with methanol/water (7:3, *v*/*v*). After vortexing and centrifugation at 12,000× *g* for 10 min at 4 °C, the supernatant was collected for LC-MS analysis.

Metabolite detection was performed using a UHPLC system coupled to a Q Exactive Orbitrap mass spectrometer (Thermo Fisher Scientific, USA) and equipped with an ACQUITY UPLC HSS T3 column (2.1 × 100 mm, 1.8 μm; Waters Corporation, Milford, MA, USA). Data were acquired in both positive and negative ionization modes. To monitor analytical stability and reproducibility, pooled quality control samples were prepared by pooling equal-volume aliquots from all samples and injected at regular intervals throughout the analytical run. Raw data were processed using Compound Discoverer 3.1 (Thermo Fisher Scientific, Waltham, MA, USA) for peak extraction, alignment, noise filtering, normalization, and peak annotation. Metabolites were annotated against the HMDB, KEGG, and mzCloud databases [14,15].

### 2.4. Differential Metabolite Identification and Pathway Analysis

Normalized metabolite abundance data were used for differential metabolite analysis between the HT and HS groups. Principal component analysis (PCA) was performed to evaluate the overall distribution of samples and the stability of the metabolomic dataset. Supervised multivariate analysis was conducted using SIMCA 14.1 to calculate variable importance in projection (VIP) values for each detected metabolite. Differential metabolites were screened using the combined criteria of VIP ≥ 1.0, *p* ≤ 0.05, and |log2FC| ≥ 1.0. The identified differential metabolites were annotated to metabolic pathways using the Kyoto Encyclopedia of Genes and Genomes (KEGG) database [15]. Pathway enrichment analysis was performed to determine significantly enriched metabolic pathways among the differential metabolites. The enrichment results were visualized using bubble plots, with the rich factor, number of metabolites, and *p*-value used to represent pathway enrichment characteristics.

### 2.5. Receiver Operating Characteristic (ROC) Analysis of Candidate Metabolites

Receiver operating characteristic (ROC) curve analysis was performed to evaluate the ability of representative differential metabolites to discriminate between HT and HS cows. Candidate metabolites were selected based on differential abundance, annotation reliability, biological relevance, and pathway representation. The ROC curves were generated using metabolite abundance values, and the area under the curve (AUC) was calculated to assess classification performance. The 95% confidence intervals of AUC values were estimated using the DeLong method [16]. Metabolites with higher AUC values were considered to have stronger discriminatory potential for distinguishing heat tolerance phenotypes. Given the limited sample size and the use of the discovery dataset for ROC analysis, the ROC results were interpreted as exploratory evidence of discriminatory potential rather than validation of biomarkers and require validation in independent populations.

### 2.6. Metagenomic Data Source and Reanalysis of Differential Microbial Taxa

The previously generated microbial abundance matrix was used to identify differential microbial taxa between HT and HS cows [7]. Differential microbial taxa were screened using linear discriminant analysis effect size (LEfSe) [17]. The Kruskal–Wallis rank-sum test was first applied to identify taxa that differed between groups, followed by linear discriminant analysis to estimate the effect size of each taxon. Taxa with LDA score > 2.0 and *p* < 0.05 were considered differential. Taxa with clear taxonomic annotation and stable detection across samples were retained for subsequent microbe–metabolite correlation analysis.

### 2.7. Correlation Analysis Between Differential Microbes and Metabolites

Spearman’s rank correlation analysis was performed to assess associations between differential microbial taxa and representative differential metabolites. To reduce the complexity of the correlation matrix, the 116 differential metabolites were first ranked in descending order according to their VIP values. Metabolites with reliable annotation and clear representation of the major enriched metabolic pathways, including nucleotide metabolism, pyrimidine metabolism, pyruvate metabolism, thiamine metabolism, and nitrogen-related metabolism, were then retained for integration analysis, resulting in 24 representative metabolites for microbe–metabolite correlation analysis. Correlation analysis was conducted using the Hmisc package (v4.8-0) in R (v4.2.2). Results were visualized as clustered heatmaps to illustrate overall association patterns between microbes and metabolites. Correlations with *p* < 0.05 were considered significant, and pairs with |r| > 0.6 and *p* < 0.05 were regarded as strong and significant associations for focused interpretation.

### 2.8. Statistical Analysis

All statistical analyses were performed in R (v4.2.2) and related software. Differences in microbial features between groups were evaluated using the Wilcoxon rank-sum test, whereas differences in metabolite abundance were assessed using the Student’s *t*-test. A *p*-value < 0.05 was considered statistically significant.

## 3. Results

### 3.1. Global Rumen Metabolic Profiles in HT and HS Cows

**Figure 1:**
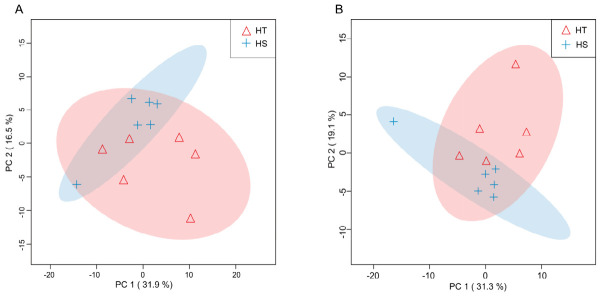
Principal component analysis (PCA) of the rumen fluid metabolome in HT and HS cows. (**A**) PCA score plot in the positive ion mode. (**B**) PCA score plot in the negative ion mode. The red and blue shaded ellipses indicate the distributions of the HT and HS groups, respectively. HT, heat-tolerant cows; HS, heat-sensitive cows.

Untargeted LC-MS metabolomic profiling was performed to characterize rumen metabolic differences between HT and HS cows. The stability and reproducibility of the LC-MS analysis were monitored using pooled quality control samples injected at regular intervals throughout the analytical run. In both positive and negative ion modes, HT and HS samples showed a tendency toward separation in the PCA score plots, suggesting that rumen metabolic profiles differed between cows with contrasting heat tolerance phenotypes (Figure 1).

In the positive ion mode, PC1 and PC2 explained 31.9% and 16.5% of the total variance, respectively. In the negative ion mode, PC1 and PC2 explained 31.3% and 19.1% of the total variance, respectively. Although some overlap was observed between the two phenotypic groups, the overall distribution indicated phenotype-associated differences in rumen metabolite composition. These results suggest that HT and HS cows possess distinct rumen metabolic backgrounds, providing a basis for subsequent differential metabolite screening and pathway enrichment analysis.

### 3.2. Differential Metabolites Between HT and HS Cows

**Figure 2:**
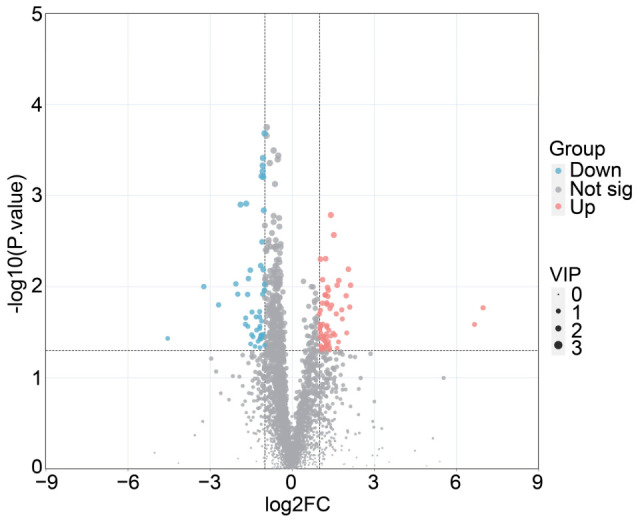
Volcano plot of differential metabolites between the HT and HS groups. Red dots indicate metabolites enriched in the HS group (Up), blue dots indicate metabolites enriched in the HT group (Down), and gray dots indicate metabolites without significant differences (Not sig). Differential metabolites were screened using variable importance in projection (VIP) values ≥ 1.0, *p* ≤ 0.05, and |log2FC| ≥ 1.0. Larger dots indicate higher VIP values.

Differential metabolite analysis was performed to identify rumen metabolites associated with heat tolerance phenotypes (Figure 2). Using the criteria of VIP ≥ 1.0, *p* ≤ 0.05, and |log2FC| ≥ 1.0, a total of 116 differential metabolites were identified between HT and HS cows (Supplementary Table S1). Among them, 66 metabolites were enriched in HS cows, whereas 50 metabolites were enriched in HT cows, indicating broad metabolic remodeling of rumen fluid between the two phenotypes.

The HS cows showed higher abundances of several nucleotide-related metabolites, including uridine 5′-monophosphate, guanosine, 2′-deoxyguanosine, 2′-deoxyadenosine, 2′-deoxyuridine, and D-ribose-5-phosphate. These metabolites suggest enhanced nucleotide turnover and stress-related metabolic activity in the rumen of HS cows. In contrast, the HT cows showed higher levels of metabolites such as thiamine, L-malate, and argininosuccinic acid, which are related to cofactor metabolism, central carbon metabolism, and nitrogen metabolism.

Overall, the differential metabolite profile indicated that HS cows were characterized mainly by nucleotide-related metabolic remodeling, whereas HT cows showed enrichment of metabolites associated with energy conversion, cofactor availability, and nitrogen metabolic balance. These results suggest that rumen metabolic adaptation differs between cows with contrasting heat tolerance phenotypes.

### 3.3. Pathway Enrichment Analysis of Differential Metabolites

**Figure 3:**
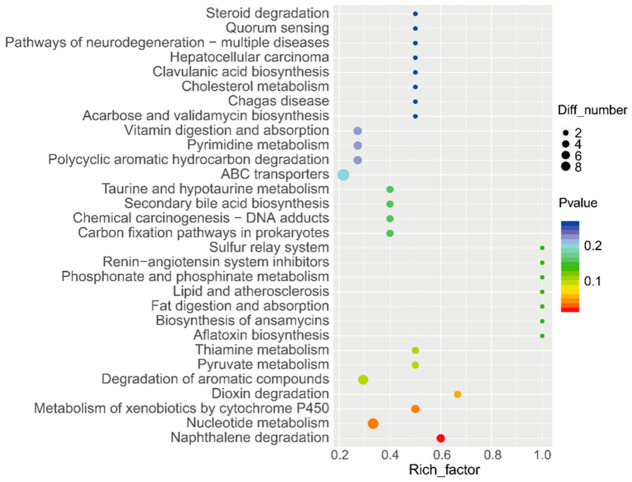
KEGG enrichment analysis of differential metabolites between HT and HS cows. Bubble size represents the number of differential metabolites enriched in each pathway, and bubble color indicates the enrichment *p*-value. The rich factor represents the ratio of the number of differential metabolites enriched in a given pathway to the total number of annotated metabolites in that pathway.

To further explore the biological relevance of the differential rumen metabolites, KEGG pathway enrichment analysis was performed [15]. The differential metabolites were mainly enriched in nucleotide metabolism, pyrimidine metabolism, pyruvate metabolism, thiamine metabolism, and ABC transporters (Figure 3).

The pathway enrichment results were consistent with the differential metabolite profile and indicated that the major metabolic differences between HT and HS cows involved nucleotide metabolism, pyrimidine metabolism, pyruvate metabolism, and thiamine metabolism. Overall, these findings suggested distinct rumen metabolic pathway profiles between HT and HS cows.

### 3.4. Differential Microbial Taxa Identified from Metagenomic Data

To explore potential links between rumen microbial variation and metabolic differences, the original metagenomic abundance matrix derived from the same rumen fluid samples was reanalyzed using LEfSe [7,17]. Using LDA > 2.0 and *p* < 0.05 as the screening criteria, 12 taxa with clear taxonomic annotation and stable detection across samples were retained for downstream integration analysis (Table 1). Among them, *Clostridium*, *Ruminococcus*, *Eubacterium*, *Bacteroides*, *Parabacteroides*, *Anaerotruncus*, *Lachnoclostridium*, *Butyricicoccus*, *Acetivibrio thermocellus*, and *Desulfobulbus oralis* showed higher abundance in HS cows, whereas *Prevotella* and *Ruminococcus flavefaciens* showed higher abundance in HT cows. These data indicate clear differences in rumen microbial composition between HT and HS cows and provide a basis for subsequent microbe–metabolite association analysis.

### 3.5. Correlation Patterns Between Differential Microbial Taxa and Representative Differential Metabolites

**Figure 4:**
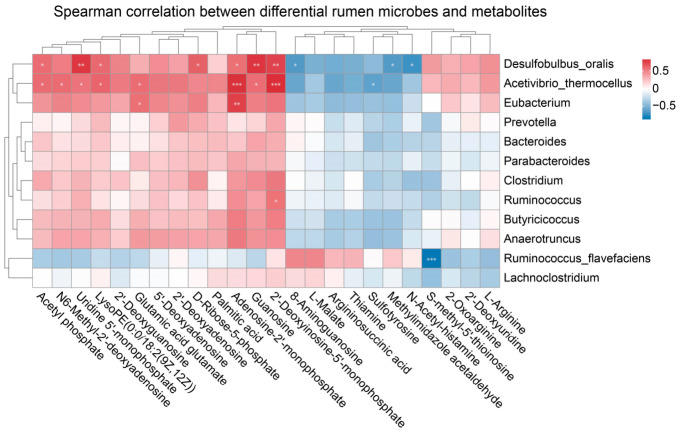
Spearman correlation heatmap between differential rumen microbes and representative differential metabolites. The heatmap shows the correlation patterns between 12 differential microbial taxa and 24 representative differential metabolites. Red indicates positive correlations, and blue indicates negative correlations. Asterisks indicate statistical significance (* *p* < 0.05, ** *p* < 0.01, *** *p* < 0.001).

Based on the 12 differential microbial taxa described in Table 1, the 116 differential metabolites were first ranked according to their VIP values, and metabolites with reliable annotation and representation of the major enriched pathways were retained for microbe–metabolite correlation analysis. This filtering process resulted in 24 representative metabolites. A microbe–metabolite correlation matrix was then constructed and analyzed using Spearman’s rank correlation analysis, and significant pairs are listed in Supplementary Table S2. The correlation heatmap showed that several HS-enriched taxa clustered with nucleotide-related metabolites, whereas HT-associated taxa tended to cluster with metabolites related to central carbon metabolism and cofactor metabolism (Figure 4).

Specifically, *Desulfobulbus oralis*, *Acetivibrio thermocellus*, and *Eubacterium* were generally positively correlated with several HS-enriched representative metabolites but negatively correlated with HT-enriched representative metabolites. In contrast, *Ruminococcus flavefaciens*, which was enriched in HT cows, showed an opposite correlation pattern. Overall, the correlation results revealed phenotype-associated microbe–metabolite association patterns in rumen fluid from HT and HS cows, indicating coordinated variation between microbial taxa and metabolites rather than direct causal relationships.

### 3.6. Evaluation of Candidate Rumen Metabolites by ROC Analysis

**Figure 5:**
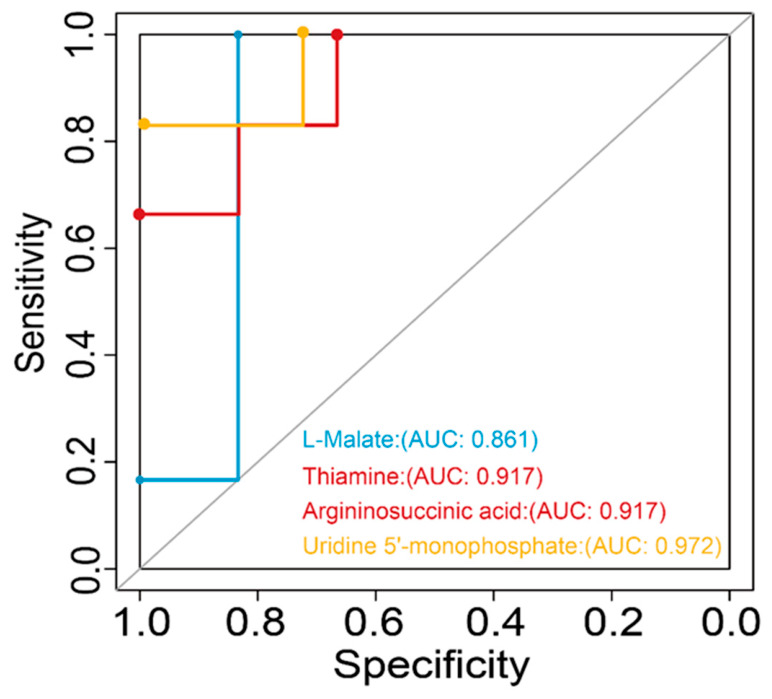
ROC curves of four representative candidate metabolites for discriminating between HT and HS cows. The candidate metabolites included Uridine 5′-monophosphate, Thiamine, L-Malate, and Argininosuccinic acid, and their corresponding AUC values were 0.972, 0.917, 0.861, and 0.917, respectively.

To assess the discriminatory potential of key differential metabolites for differentiating heat tolerance phenotypes, ROC analysis was performed on four representative metabolites. Uridine 5′-monophosphate showed the highest AUC (0.972), while thiamine and argininosuccinic acid each showed an AUC of 0.917, and L-malate showed an AUC of 0.861 (Figure 5).

These results suggest that these four metabolites showed discriminatory potential in the current discovery dataset and may serve as exploratory candidate metabolic indicators for distinguishing HT and HS cows. However, given the small sample size and the use of the same dataset for metabolite discovery and ROC analysis, these metabolites should not be considered validated biomarkers at this stage. Their robustness and generalizability still need to be validated in larger and independent cohorts.

## 4. Discussion

Heat stress affects dairy cows through changes in feed intake, rumen fermentation, endocrine regulation, immune function, and systemic metabolism [1,2,3,4,5,6]. In the present study, rumen fluid metabolomics was integrated with previously generated metagenomic abundance data from the same HT and HS cows to explore rumen microbe–metabolite features associated with heat tolerance [7]. The results revealed differences in rumen metabolic profiles, enriched pathways, microbial taxa, and microbe–metabolite association patterns between HT and HS cows, suggesting that rumen metabolic remodeling and its microbial context may be involved in natural variation in heat tolerance.

HS cows showed higher abundances of nucleotide-related metabolites, including uridine 5′-monophosphate, guanosine, 2′-deoxyguanosine, 2′-deoxyadenosine, 2′-deoxyuridine, and D-ribose-5-phosphate. Consistently, KEGG enrichment highlighted nucleotide and pyrimidine metabolism [15]. This pattern may reflect enhanced nucleotide turnover and altered nucleotide metabolism in the rumen of HS cows. Under heat stress, reduced feed intake, altered rumen fermentation, oxidative stress, and changes in rumen microbial composition can induce broad metabolic remodeling in dairy cows [2,3,5,6,18,19,20]. The increased levels of deoxynucleosides, such as 2′-deoxyguanosine, 2′-deoxyadenosine, and 2′-deoxyuridine, may indicate changes in DNA-related nucleotide turnover, whereas uridine 5′-monophosphate and guanosine may reflect altered pyrimidine and purine metabolism. In addition, D-ribose-5-phosphate is an important intermediate linking the pentose phosphate pathway with PRPP formation and nucleotide biosynthesis [21,22]. Therefore, the enrichment of nucleotide-related metabolites in HS cows may represent a rumen metabolic state characterized by increased microbial and biochemical turnover under heat stress. This interpretation is consistent with our previous metagenomic study, in which HS cows showed enrichment of pathways related to the TCA cycle, pyruvate metabolism, and purine metabolism [7].

In contrast, HT cows had higher levels of thiamine, L-malate, and argininosuccinic acid. These metabolites may collectively reflect a rumen metabolic state that better supports central carbon metabolism, cofactor availability, and nitrogen-related metabolic balance under heat stress. L-malate is an intermediate of central carbon metabolism and has been reported to affect mixed ruminal microbial fermentation [15,23,24]. In addition, malate can stimulate lactate utilization by *Selenomonas ruminantium*, suggesting a potential role in supporting rumen fermentation stability [25]. Thus, higher L-malate in HT cows may be associated with more favorable rumen carbon flux and fermentation conditions. Thiamine is an essential cofactor for carbohydrate metabolism, and rumen microbial synthesis is an important source of thiamine in dairy cows [26,27]. Previous studies have shown that thiamine supplementation can modulate rumen fermentation, carbohydrate metabolism, and bacterial community structure under high-grain or rumen-challenge conditions [26,27,28]. Therefore, the higher thiamine level in HT cows may indicate better maintenance of microbial cofactor availability and carbohydrate-utilization capacity during heat exposure. Argininosuccinic acid is an intermediate involved in arginine biosynthesis and urea-cycle-related metabolism [14,15,29]. Because arginine metabolism is closely linked to nitrogen disposal, nitric oxide production, polyamine synthesis, and cellular metabolic regulation [30], the enrichment of argininosuccinic acid in HT cows may indicate differences in nitrogen utilization or amino acid-related metabolic balance. However, the direct causal relationship between these metabolites and heat tolerance remains to be validated.

The reanalysis of metagenomic data identified 12 differential microbial taxa [7,17], indicating that the metabolic differences between HT and HS cows occurred together with distinct rumen microbial backgrounds. Most selected taxa were enriched in HS cows, whereas *Prevotella* and *R. flavefaciens* were enriched in HT cows. *Prevotella* is involved in ruminal protein and polysaccharide utilization as well as propionate production [31], and *R. flavefaciens* is a well-known cellulolytic bacterium involved in plant fiber degradation [32,33,34,35,36,37]. In the context of the present metabolomic results, the enrichment of these HT-associated taxa is consistent with the higher levels of metabolites related to carbon metabolism and cofactor availability, such as L-malate and thiamine. These findings suggest that the metabolic differences between HT and HS cows were accompanied by distinct rumen microbial features, providing a microbial context for the subsequent microbe–metabolite correlation analysis.

Correlation analysis further linked differential microbial taxa with representative metabolites. The HS-enriched taxa, including *D. oralis*, *A. thermocellus*, and *Eubacterium*, were generally positively associated with HS-enriched nucleotide-related metabolites, but negatively associated with HT-enriched metabolites such as L-malate, thiamine, and argininosuccinic acid. In contrast, the HT-enriched *R. flavefaciens* showed the opposite pattern. These coordinated associations suggest that the metabolic differences between HT and HS cows were accompanied by distinct rumen microbial features. This finding is consistent with previous multi-omics studies indicating that rumen microbial and metabolic profiles are closely linked to dairy cow performance and heat stress responses [12,13,19,20,38]. However, these correlation-based results do not establish causality.

Taken together, the correlation-based integration of rumen metabolomics and metagenomic abundance data provided a more comprehensive interpretation of the phenotype-associated rumen differences between HT and HS cows. The HS group was characterized by nucleotide-related metabolic changes together with several HS-enriched microbial taxa, whereas the HT group showed higher levels of L-malate, thiamine, and argininosuccinic acid, accompanied by HT-enriched taxa such as *R. flavefaciens*. These coordinated patterns suggest that natural heat tolerance in dairy cows may be associated with a distinct rumen microbe–metabolite environment, rather than with isolated changes in individual metabolites or microbial taxa alone. Although these associations require validation in larger independent cohorts, they provide candidate microbial and metabolic links for future studies on rumen-mediated heat tolerance.

The ROC analysis identified uridine 5′-monophosphate, thiamine, L-malate, and argininosuccinic acid as candidate rumen metabolites for distinguishing HT and HS cows. Similar metabolomics-based strategies have been used to identify heat stress-related biomarkers in dairy cows [18,39,40,41,42]. However, because the ROC analysis was based on the same small cohort used for discovery, these metabolites should be considered exploratory candidates and require validation in larger independent populations [43].

## 5. Conclusions

In conclusion, this study integrated rumen metabolomics with metagenomic abundance data to characterize microbe–metabolite patterns associated with heat tolerance in dairy cows. HS cows were mainly characterized by nucleotide-related metabolic remodeling, whereas HT cows showed enrichment of metabolites related to central carbon and cofactor metabolism. These findings extend our previous rumen metagenomic work by adding newly generated rumen metabolomic evidence and provide exploratory candidate metabolic indicators and microbe–metabolite associations for future validation in dairy cattle.

## Figures and Tables

**Table 1:** Twelve key differential microbial taxa identified by LEfSe.

Taxon	Taxonomic Level	Enriched Group	LDA Score	HT Relative Abundance, %	HS Relative Abundance, %
*Clostridium*	Genus	HS	4.704	2.6755 ± 1.6557	2.7791 ± 0.6030
*Ruminococcus*	Genus	HS	4.365	1.4224 ± 0.6903	1.4609 ± 0.3702
*Eubacterium*	Genus	HS	4.319	0.5782 ± 0.2596	0.8276 ± 0.2353
*Bacteroides*	Genus	HS	4.189	0.7644 ± 0.2596	0.8467 ± 0.1845
*Parabacteroides*	Genus	HS	3.381	0.1206 ± 0.0423	0.1304 ± 0.0359
*Anaerotruncus*	Genus	HS	3.337	0.0677 ± 0.0326	0.0891 ± 0.0262
*Lachnoclostridium*	Genus	HS	3.315	0.1267 ± 0.0382	0.1301 ± 0.0652
*Butyricicoccus*	Genus	HS	3.253	0.0608 ± 0.0271	0.0787 ± 0.0208
*Acetivibrio_thermocellus*	Species	HS	2.550	0.0018 ± 0.0006	0.0029 ± 0.0010
*Desulfobulbus oralis*	Species	HS	2.456	0.0005 ± 0.0003	0.0026 ± 0.0041
*Prevotella*	Genus	HT	4.979	5.9430 ± 3.2391	4.9369 ± 1.2261
*Ruminococcus_flavefaciens*	Species	HT	3.905	0.3535 ± 0.1841	0.1896 ± 0.0392

Note: Differential taxa were identified using LEfSe with LDA score > 2.0 and *p* < 0.05. For each sample, the relative abundance of each taxon was calculated as the abundance value of that taxon divided by the total taxonomic abundance of the corresponding sample and multiplied by 100. Values are presented as mean ± SD, *n* = 6 per group.

## Data Availability

The original contributions presented in this study are included in the article/Supplementary Materials. Further inquiries can be directed to the corresponding author.

## Supplementary Materials

The following supporting information can be downloaded at: https://www.mdpi.com/article/10.3390/ani16142152/s1, Table S1: Differential rumen metabolites between heat-tolerant (HT) and heat-sensitive (HS) cows; Table S2: Significant Spearman correlations between differential microbial taxa and representative differential rumen metabolites.

## Abbreviations

The following abbreviations are used in this manuscript:

HT	heat-tolerant
HS	heat-sensitive
TOPSIS	Technique for Order Preference by Similarity to Ideal Solution
RT	rectal temperature
RR	respiratory rate
SI	salivation index
HSP70	heat shock protein 70
LC-MS	liquid chromatography–mass spectrometry
PCA	principal component analysis
VIP	variable importance in projection
KEGG	Kyoto Encyclopedia of Genes and Genomes
ROC	receiver operating characteristic
AUC	area under the curve
LEfSe	linear discriminant analysis effect size
LDA	linear discriminant analysis

## References

[1] Kadzere C.T., Murphy M.R., Silanikove N., Maltz E. (2002). Heat stress in lactating dairy cows: A review. Livest. Prod. Sci..

[2] Wheelock J.B., Rhoads R.P., VanBaale M.J., Sanders S.R., Baumgard L.H. (2010). Effects of heat stress on energetic metabolism in lactating Holstein cows. J. Dairy Sci..

[3] Baumgard L.H., Rhoads R.P. (2013). Effects of heat stress on postabsorptive metabolism and energetics. Annu. Rev. Anim. Biosci..

[4] Becker C.A., Collier R.J., Stone A.E. (2020). Invited review: Physiological and behavioral effects of heat stress in dairy cows. J. Dairy Sci..

[5] Kim S.H., Ramos S.C., Valencia R.A., Cho Y.I., Lee S.S. (2022). Heat Stress: Effects on Rumen Microbes and Host Physiology, and Strategies to Alleviate the Negative Impacts on Lactating Dairy Cows. Front. Microbiol..

[6] Park T., Ma L., Gao S., Bu D., Yu Z. (2022). Heat stress impacts the multi-domain ruminal microbiota and some of the functional features independent of its effect on feed intake in lactating dairy cows. J. Anim. Sci. Biotechnol..

[7] Li M., Wang Z., Ma Z., Wang Y., Jia H., Zhang L., Chen P., Mao Y., Yang Z. (2025). Metagenomic analysis reveals microbial drivers of heat resistance in dairy cattle. Anim. Microbiome.

[8] Bickhart D.M., Weimer P.J. (2018). Symposium review: Host–rumen microbe interactions may be leveraged to improve the productivity of dairy cows. J. Dairy Sci..

[9] Loor J.J., Elolimy A.A., McCann J.C. (2016). Dietary impacts on rumen microbiota in beef and dairy production. Anim. Front..

[10] Guo H., Zhou G., Tian G., Liu Y., Dong N., Li L., Zhang S., Chai H., Chen Y., Yang Y. (2021). Changes in Rumen Microbiota Affect Metabolites, Immune Responses and Antioxidant Enzyme Activities of Sheep under Cold Stimulation. Animals.

[11] Kim D.H., Kim M.H., Kim S.B., Son J.K., Lee J.H., Joo S.S., Gu B.H., Park T., Park B.Y., Kim E.T. (2020). Differential Dynamics of the Ruminal Microbiome of Jersey Cows in a Heat Stress Environment. Animals.

[12] Zhang C., Wang M., Liu H., Jiang X., Chen X., Liu T., Yin Q., Wang Y., Deng L., Yao J. (2023). Multi-omics reveals that the host-microbiome metabolism crosstalk of differential rumen bacterial enterotypes can regulate the milk protein synthesis of dairy cows. J. Anim. Sci. Biotechnol..

[13] Jiang B., Qin C., Xu Y., Song X., Fu Y., Li R., Liu Q., Shi D. (2024). Multi-omics reveals the mechanism of rumen microbiome and its metabolome together with host metabolome participating in the regulation of milk production traits in dairy buffaloes. Front. Microbiol..

[14] Wishart D.S., Guo A., Oler E., Wang F., Anjum A., Peters H., Dizon R., Sayeeda Z., Tian S., Lee B.L. (2022). HMDB 5.0: The Human Metabolome Database for 2022. Nucleic Acids Res..

[15] Kanehisa M., Furumichi M., Sato Y., Ishiguro-Watanabe M., Tanabe M. (2021). KEGG: Integrating viruses and cellular organisms. Nucleic Acids Res..

[16] DeLong E.R., DeLong D.M., Clarke-Pearson D.L. (1988). Comparing the areas under two or more correlated receiver operating characteristic curves: A nonparametric approach. Biometrics.

[17] Segata N., Izard J., Waldron L., Gevers D., Miropolsky L., Garrett W.S., Huttenhower C. (2011). Metagenomic biomarker discovery and explanation. Genome Biol..

[18] Yue S., Ding S., Zhou J., Yang C., Hu X., Zhao X., Wang Z., Wang L., Peng Q., Xue B. (2020). Metabolomics approach explore diagnostic biomarkers and metabolic changes in heat-stressed dairy cows. Animals.

[19] Feng L., Zhang Y., Liu W., Du D., Jiang W., Wang Z., Li N., Hu Z. (2023). Altered rumen microbiome and correlations of the metabolome in heat-stressed dairy cows at different growth stages. Microbiol. Spectr..

[20] Wang Z., Liu L., Pang F., Zheng Z., Teng Z., Miao T., Fu T., Rushdi H.E., Yang L., Gao T. (2022). Novel insights into heat tolerance using metabolomic and high-throughput sequencing analysis in dairy cow rumen fluid. Animal.

[21] Lane A.N., Fan T.W. (2015). Regulation of mammalian nucleotide metabolism and biosynthesis. Nucleic Acids Res..

[22] Hove-Jensen B., Andersen K.R., Kilstrup M., Martinussen J., Switzer R.L., Willemoës M. (2017). Phosphoribosyl Diphosphate (PRPP): Biosynthesis, Enzymology, Utilization, and Metabolic Significance. Microbiol. Mol. Biol. Rev. MMBR.

[23] Martin S.A., Streeter M.N. (1995). Effect of malate on in vitro mixed ruminal microorganism fermentation. J. Anim. Sci..

[24] Martin S.A., Sullivan H.M., Evans J.D. (2000). Effect of Sugars and Malate on Ruminal Microorganisms. J. Dairy Sci..

[25] Evans J.D., Martin S.A. (1997). Factors affecting lactate and malate utilization by Selenomonas ruminantium. Appl. Environ. Microbiol..

[26] Pan X.H., Yang L., Xue F.G., Xin H.R., Jiang L.S., Xiong B.H., Beckers Y. (2016). Relationship between thiamine and subacute ruminal acidosis induced by a high-grain diet in dairy cows. J. Dairy Sci..

[27] Pan X., Xue F., Nan X., Tang Z., Wang K., Beckers Y., Jiang L., Xiong B. (2017). Illumina sequencing approach to characterize thiamine metabolism-related bacteria and the impacts of thiamine supplementation on ruminal microbiota in dairy cows fed high-grain diets. Front. Microbiol..

[28] Zhao Y., Xue F., Hua D., Wang Y., Pan X., Nan X., Sun F., Jiang L., Xiong B. (2020). Metagenomic insights into effects of thiamine supplementation on carbohydrate-active enzymes’ profile in dairy cows fed high-concentrate diets. Animals.

[29] Husson A., Brasse-Lagnel C., Fairand A., Renouf S., Lavoinne A. (2003). Argininosuccinate synthetase from the urea cycle to the citrulline-NO cycle. Eur. J. Biochem..

[30] Morris S.M. (2016). Arginine Metabolism Revisited. J. Nutr..

[31] Betancur-Murillo C.L., Aguilar-Marín S.B., Jovel J. (2023). Prevotella: A key player in ruminal metabolism. Microorganisms.

[32] Hua D., Hendriks W.H., Xiong B., Pellikaan W.F. (2022). Starch and cellulose degradation in the rumen and applications of metagenomics on ruminal microorganisms. Animals.

[33] Koike S., Kobayashi Y. (2009). Fibrolytic rumen bacteria: Their ecology and functions. Asian-Australas. J. Anim. Sci..

[34] Weimer P.J. (2022). Degradation of cellulose and hemicellulose by ruminal microorganisms. Microorganisms.

[35] Miron J., Ben-Ghedalia D., Morrison M. (2001). Invited Review: Adhesion Mechanisms of Rumen Cellulolytic Bacteria. J. Dairy Sci..

[36] Berg Miller M.E., Antonopoulos D.A., Rincon M.T., Band M., Bari A., Akraiko T., Hernandez A., Thimmapuram J., Henrissat B., Coutinho P.M. (2009). Diversity and strain specificity of plant cell wall degrading enzymes revealed by the draft genome of Ruminococcus flavefaciens FD-1. PLoS ONE.

[37] Dassa B., Borovok I., Ruimy-Israeli V., Lamed R., Flint H.J., Duncan S.H., Henrissat B., Coutinho P., Morrison M., Mosoni P. (2014). Rumen cellulosomics: Divergent fiber-degrading strategies revealed by comparative genome-wide analysis of six ruminococcal strains. PLoS ONE.

[38] Xue M.-Y., Sun H.-Z., Wu X.-H., Liu J.-X., Guan L.L. (2020). Multi-omics reveals that the rumen microbiome and its metabolome together with the host metabolome contribute to individualized dairy cow performance. Microbiome.

[39] Tian H., Zheng N., Wang W., Cheng J., Li S., Zhang Y., Wang J. (2016). Integrated metabolomics study of the milk of heat-stressed lactating dairy cows. Sci. Rep..

[40] Fan C.-Y., Su D., Tian H., Hu R.-T., Ran L., Yang Y., Su Y.-J., Cheng J.-B. (2019). Milk production and composition and metabolic alterations in the mammary gland of heat-stressed lactating dairy cows. J. Integr. Agric..

[41] Jorge-Smeding E., Leung Y.H., Ruiz-González A., Xu W., Astessiano A.L., Trujillo A.I., Rico D.E., Kenéz Á. (2024). Plasma and milk metabolomics revealed changes in amino acid metabolism in Holstein dairy cows under heat stress. Animal.

[42] Li M., Wang Z., Zhu L., Wang Y., Zhang L., Jia H., Yang Z., Karrow N.A., Mao Y. (2025). Integrative blood transcriptomic and metabolomic profiling reveals biomarkers of natural heat tolerance in Holstein cows. J. Dairy Sci..

[43] Xia J., Broadhurst D.I., Wilson M., Wishart D.S. (2013). Translational biomarker discovery in clinical metabolomics: An introductory tutorial. Metabolomics.

